# Cholesterol Granuloma in Odontogenic Cyst: An Enigmatic Lesion

**DOI:** 10.1155/2016/6105142

**Published:** 2016-12-13

**Authors:** Mala Kamboj, Anju Devi, Shruti Gupta

**Affiliations:** Department of Oral Pathology and Microbiology, Postgraduate Institute of Dental Sciences, Pt. BD Sharma University of Medical Sciences, Rohtak, Haryana, India

## Abstract

Cholesterol granuloma (CG) is the outcome of the foreign body type of response to the accumulation of cholesterol crystals and is frequently present in conjunction with chronic middle ear diseases. Recently, cases of CG in jaws have been reported, but still, very few cases have been found of CG in dental literature. This article presents three rare cases of CG in the wall of odontogenic cysts emphasizing on its possible role in expansion of the associated lesion and bone erosion. It also lays stress on the fact that more cases of CG should be reported so that its nature and pathogenesis in the oral cavity become more perceivable.

## 1. Introduction

Cholesterol granuloma (CG) is a histopathological entity that is characterized by collection of numerous cholesterol clefts, which are associated with foreign body giant cells, foam cells, and hemosiderin filled macrophages [1]. Most common site of occurrence of CG is middle ear (generally associated with chronic middle ear diseases). Lungs, brain, kidneys, mastoid process, breast, sella turcica, pontocerebelline angle, testis, and apex of temporal bone pyramid are the other sites where CG can occur [2]. Recently, it was reported that CG could also occur in the facial skeleton, maxillary antrum and frontal bone being the two common sites [3]. The clinical symptoms are nonspecific and depend on the localization and extent in each individual case [1]. Very few cases of CG occurring in jaws have been reported in the English literature. We report three unique cases where the odontogenic cysts were secondarily inflamed and showed expansion and associated bone destruction due to CG formation.

## 2. Case Series

### 2.1. Case  1

**Figure 1:**
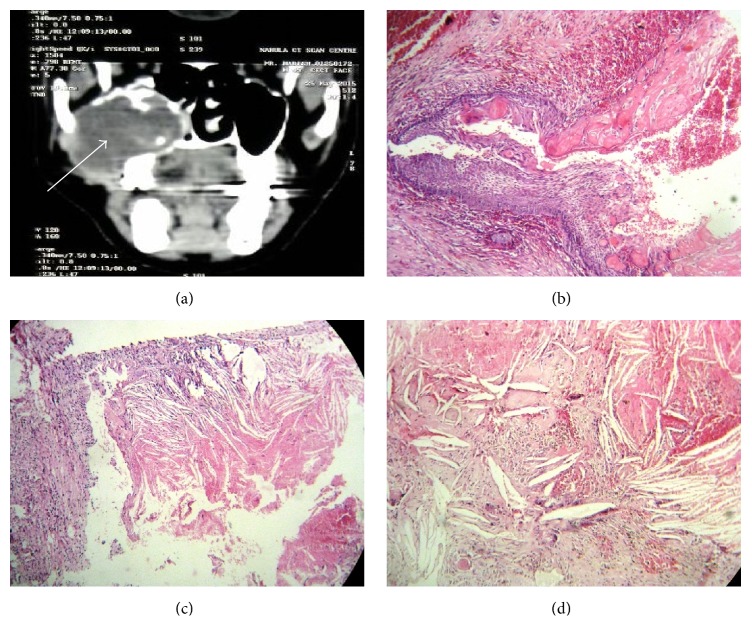
(a) CT scan image showing huge expansile, well demarcated, and osteolytic mass extending from maxilla into the maxillary sinus on right side; (b) proliferation of cystic odontogenic epithelial lining with palisaded hyperchromatic columnar nuclei, stellate reticulum like areas, and sheets of abundant ghost cells (H/E 4x); (c) cholesterol clefts in cystic wall (H/E 4x); (d) cholesterol clefts in cystic wall (H/E 10x).

A 45-year-old male reported to the institute with the complaint of swelling in the posterior tooth region of right maxilla since one month. Patient gave a history for the extraction of 17, 18 (with grade II mobility) for the same in private dental clinic but after extraction the swelling persisted. On palpation, swelling was soft and fluctuant in nature extending from the buccal vestibule till midline of the hard palate. FNAC was attempted but the mass bled profusely. CT scan revealed a huge expansile, well demarcated, and osteolytic mass extending from maxilla into the maxillary sinus. A provisional diagnosis of keratocystic odontogenic tumor (KCOT) was made. Surgical enucleation of the cyst was performed and the tissue was sent for histopathological examination. The sections revealed proliferation of cystic odontogenic epithelial lining with palisaded hyperchromatic columnar nuclei, stellate reticulum like areas, and sheets of abundant ghost cells. Surrounding connective tissue stroma showed mature collagen bundles. Abundant hemorrhagic areas and fibrin deposits were evident both in fibrous wall and in cystic lumen. Also visible both in the connective tissue and in cystic lumen were abundant cholesterol clefts and hemorrhagic areas. Focal areas of ghost cell formation were also visible in the stroma surrounded by foreign body giant cells and hemosiderin pigmentation. Thus, based on this a final diagnosis of* calcifying odontogenic cyst with cholesterol granuloma* was made (Figure 1).

### 2.2. Case  2

**Figure 2:**
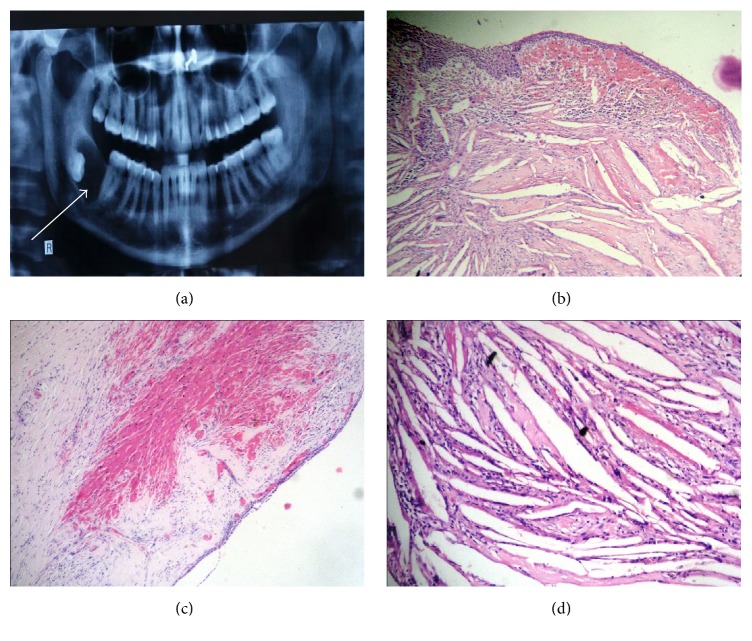
(a) An OPG showing a well-defined radiolucency in relation to crown of 48; (b) nonkeratinized stratified squamous cystic epithelial lining which showed proliferation in few areas associated with underlying cholesterol clefts and extravasated RBCs in the cystic wall (H/E 4x); (c) cystic lining with abundant area of hemorrhage in the cystic wall (H/E 4x); (d) cholesterol clefts in cystic wall (H/E 20x).

A 38-year-old female complained of swelling since 6 months at right side posterior mandible. Patient stated that the swelling gradually increased in size with pus discharge and no pain. Intraorally, the swelling was 3 × 2 cm in size at right angle region with missing 48. Overlying mucosa appeared smooth, nonerythematous and nontender on palpation. A well-defined radiolucency in relation to crown of 48 was seen in the orthopantomography. A provisional diagnosis of dentigerous cyst and keratocystic odontogenic tumor (KCOT) was made. The cyst was surgically enucleated and the histopathology revealed nonkeratinized stratified squamous cystic epithelial lining, 2-3-cell layer thick, which showed proliferation in few areas associated with underlying inflammation. In the connective tissue cystic capsule, mixed inflammatory cell infiltrates with areas of hemorrhage were evident. A part of the tissue revealed abundant cholesterol clefts in the cystic capsule in association with multinucleated giant cells. Based on this a diagnosis of* dentigerous cyst with cholesterol granuloma* was made (Figure 2).

### 2.3. Case  3

**Figure 3:**
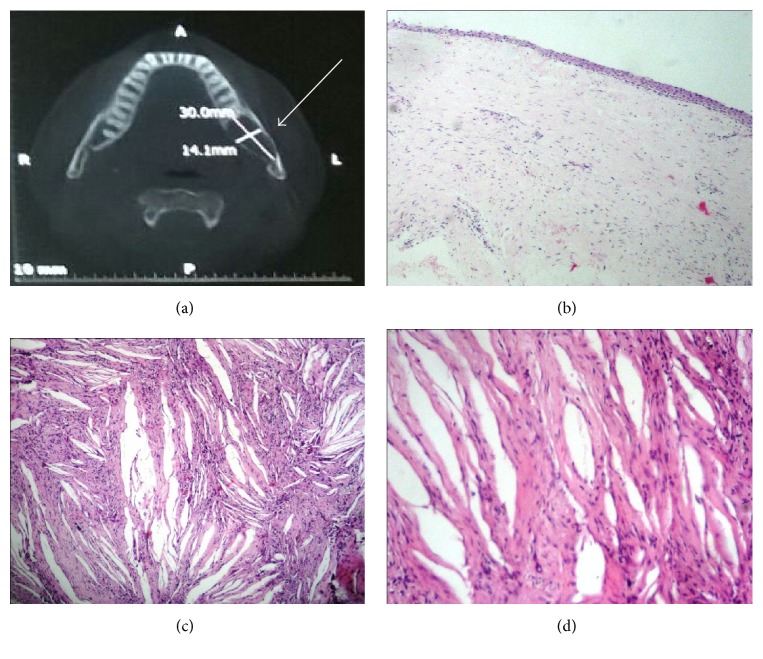
(a) CT scan revealed a well-defined radiolucency (30 × 14 mm in size) present on left angle of mandible region; (b) on incisional biopsy, 2-3-cell layer thick nonkeratinized stratified squamous cystic epithelial lining which showed proliferation in few areas associated with underlying inflammation (H/E 4x); (c) on excisional biopsy cholesterol clefts in cystic wall (H/E 10x); (d) cholesterol clefts in cystic wall (H/E 40x).

A 47-year-old male reported with complaint of pain in lower left posterior mandible for past two days. He gave history of swelling on this side since two months. Mild swelling was present on left side of mandible, which was hard on palpation and nontender. Intraorally the swelling was soft in consistency and painful with a slight opening present on crest region through which yellowish green color discharge was noticed. Computed tomography revealed a well-defined radiolucency (30 × 14 mm in size) present on left angle of mandible region with 38 displaced near the inferior border of the mandible. Incisional biopsy was sent for histopathological evaluation which revealed cystic lining of varying thickness, nonkeratinized stratified squamous in nature with few areas of mucus metaplasia. Connective tissue capsule was fibrocellular with vascular channels and mild inflammatory cell infiltrate and a diagnosis of dentigerous cyst was made. After this the excised tissue was sent which after repeated regrossing and tissue sectioning did not show any cystic lining. The connective tissue stroma was ectomesenchymal and fibrocellular with abundant cholesterol clefts and chronic inflammatory cell infiltrate (Figure 3).

## 3. Discussion

CG histopathologically depicts a large collection of longitudinal cholesterol clefts that are formed at the site of cholesterol crystals because of dissolution of crystals at the time of tissue processing and is embedded in fibrous granulation tissue with surrounding foreign body type of multinucleated giant cells and macrophages filled with hemosiderin [4]. After an extensive survey, six cases of CG have been found in the oral cavity, in which only three cases of CG occurring in the wall of odontogenic cyst have been reported so far [3–7]. We report additional three cases of CG occurring in wall of odontogenic cysts (Table 1).

An ambiguity has always persisted regarding the terminology of CG in the oral cavity, as Bhaskar, Wood, and Goaz described a lesion in the jaw with similar histopathological features but termed it cholesteatoma [3]. Stating that cholesterol was the main component of tumor the term “cholesteatoma” was introduced in 1838 by Muller [7]. Cholesteatoma describes those cystic cavities lined by keratinized squamous epithelium and surrounded by stroma of variable thickness. The microscopic diagnosis depends entirely on the presence and identification of squamous epithelium and/or laminated keratinized material [3]. The main difference between histogenesis of CG and cholesteatoma is that no epithelium is involved in formation of CG. Transition of CG into cholesteatomas has not been observed, although the two anomalies may occur simultaneously [7]. Thus, use of the term cholesteatoma for a mass of connective tissue with numerous cholesterol crystals but without epithelial components and keratin is inappropriate and misleading [3].

CG should be considered in clinical-radiographic differential diagnoses of odontogenic cysts or tumors. Netto et al. reported a case of mandibular CG mimicking a dentigerous cyst [8].

The cholesterol crystals are reported to occur more commonly in inflammatory cysts, especially in radicular cysts. Lowest incidence was reported in noninflammatory cysts such as odontogenic keratocyst [5]. In all of our cases, CG is seen in the wall of cysts with developmental background.

The pathogenesis of CG is controversial as many possible mechanisms have been proposed to explain it but no clear consensus has been made. CG is formed due to irritant effect of accumulated cholesterol crystals as a result of the breakdown of blood, inflammatory tissue, or exudate. They attract foreign body giant cells and thus cause fibrosis [7]. Our cases also seem to reflect similar mechanism. In the middle ear, formation of CG could be attributed to the obstruction of ear drainage. Secondary to absorption of air into mucosa a negative pressure is created within the air cavity because of disturbance of air drainage. As a result, mucosal edema and hemorrhage develop. Hematoma from the mucosal bleeding would not be absorbed, resulting in its conversion to cholesterol crystals. However this cannot be a possible cause of CG occurring in the mandible due to the absence of intrabony cavities or drainage pathways [4].

The buildup of cholesterol crystals in the cyst wall and cystic fluid could result from the disintegrating red blood cells of stagnant blood vessels within the lesion, circulating plasma lipids, or fatty degeneration of connective tissue in a cavity blocked by inflammation [6]. Our cases reported abundant areas of hemorrhage, which could be the cause of formation of CG. However, little is currently known about the molecular mechanism for CG formation in the cyst wall. A recent study by Yamazaki et al. suggested that formation of CG could be related to the presence of abundant perlecan (a basement membrane heparan sulfate proteoglycan) in the cyst wall of immature granulation tissue [9].

CG could lead to expansion of the lesion with which it is associated. Almada et al. [10] reported that there are chances of primarily or secondarily inflamed odontogenic lesions that exhibit foreign body reaction to cholesterol crystals in their capsule and could extend to maxillary sinuses due to anatomical continuity. Yamazaki et al. [9] reported that CG seems to be one of the driving forces for growth of jaw cysts, especially those with inflammatory background. They suggested that low density lipoprotein entrapped by perlecan is accumulated and oxidized in the extracellular space and that oxidized-low density lipoprotein is scavenged by macrophages and is primarily deposited intracellularly; then the macrophages are converted into lipid-laden foamy cells. These foamy cells may originally rupture and release lipids concentrated in their cytoplasm into the extracellular space. Following this, concentrated free cholesterol results in crystallization. Cholesterol crystals in turn cause foreign body reactions to extend inflammatory reactions for cystic growth. Bone erosion may be seen in cholesterol granuloma showing expansive growth [1]. Nair PN et al. stated that macrophages change the hydrophobic cholesterol crystals into a soluble form by incorporating it into lipoprotein vehicle. However, the large cholesterol crystals resist internalization by macrophages and circumfuse to form multinucleated giant cells. Although they persist for prolonged periods, the phagocytes failed to degrade cholesterol and release inflammatory and bone resorptive mediators that cause further loss of bone and extension of lesion [6].

## 4. Conclusion

CG is considered as a nonspecific histopathological reaction to cholesterol crystals rather than a clinical or pathological entity. As its clinical and radiographic characteristics are nonspecific, it should be considered in differential diagnosis of odontogenic cyst and tumors and histopathological analysis is essential for a correct final diagnosis of CG. Due to paucity of the intraoral reported CG cases the true nature and pathogenesis are still ambiguous. There also persists confusion on the terminologies and distinctiveness of CG reported in the oral cavity and ear. More CG cases of the oral cavity should be brought to light so that their unique identity could be revealed and elaborated upon.

## Figures and Tables

**Table 1:** Reported cases of cholesterol granuloma in association with odontogenic cyst.

Author	Year	Age/sex	Site	Associated with
Lee et al. [4]	2010	68/male	Right anterior to posterior mandible	Dentigerous cyst
Bhullar et al. [5]	2012	43/male	Left posterior mandible	Dentigerous cyst
Aparna et al. [6]	2013	68/female	Right posterior mandible	Ameloblastomatous calcifying odontogenic cyst
Case 1	2016	45/Male	Right posterior maxilla	Calcifying odontogenic cyst
Case 2	2016	38/Female	Right posterior mandible	Dentigerous cyst
Case 3	2016	47/Male	Left posterior mandible	Dentigerous cyst
